# Failure of diplodiatoxin to induce diplodiosis in juvenile goats

**DOI:** 10.4102/ojvr.v87i1.1712

**Published:** 2020-03-05

**Authors:** Christo J. Botha, Louis G.J. Ackerman, Mxolisi G. Masango, Luke F. Arnot

**Affiliations:** 1 Department of Paraclinical Sciences, Faculty of Veterinary Science, University of Pretoria, Pretoria, South Africa; 2 Analytics and Institutional Research Unit, University of the Witwatersrand, Johannesburg, South Africa; 3 Department of Production Animal Studies, Faculty of Veterinary Science, University of Pretoria, Onderstepoort, South Africa

**Keywords:** diplodiosis, diplodiatoxin, *Stenocarpella maydis*, mycotoxin, neuromycotoxicosis

## Abstract

Diplodiosis is an important neuromycotoxicosis of ruminants in South Africa when grazing on harvested maize fields in winter. It is believed to be caused by mycotoxin(s) synthesised by *Stenocarpella (Diplodia) maydis*. Although several metabolites have been isolated from *S. maydis* culture material, none of these have been administered to ruminants to reproduce the disease. The objectives of this study were to isolate diplodiatoxin and to administer it to juvenile goats. Diplodiatoxin, considered as a major metabolite, was purified from *S. maydis*-infected maize cultures (Coligny 2007 isolate). Following intravenous administration of 2 mg and 4 mg diplodiatoxin/kg body weight for five consecutive days to two juvenile goats, no clinical signs reminiscent of diplodiosis were observed. Based on previous experimental results and if diplodiatoxin was the causative compound, the dosage regimen employed was seemingly appropriate to induce diplodiosis. In addition, intraruminal administration of 2 mg/kg diplodiatoxin to one goat for three consecutive days also did not induce clinical signs. It appears as if diplodiatoxin alone is not the causative compound. Other metabolites and/or mixtures of diplodiatoxin and other mycotoxins, when available in sufficient quantities, should also be evaluated.

## Introduction

*Stenocarpella* (*Diplodia*) *maydis* (Berk.) Sutton is one of the most prevalent cob and stalk rot pathogens of maize responsible for a decline in grain quality and yield (Flett & McLaren 1994). *Stenocarpella maydis*-infected maize is also associated with intoxication in ruminants, resulting in the neuromycotoxicosis known as diplodiosis (Kellerman et al. 2005). Diplodiosis is an important toxicosis in South Africa because of the fact that many farmers utilise harvested maize fields, locally called stover, as an important winter food source (Kellerman et al. 2005). The first record of diplodiosis in South Africa is reported by Van der Bijl (1914) who cites Government Veterinary Surgeon Webb who submitted maize cobs in August 1912 from Mooi River, KwaZulu-Natal province. Mr Webb wrote (Van der Bijl 1914):

I am also sending you some specimens of mealies taken from fields in which cattle have become sick, showing symptoms of intoxication and paralysis due, I believe, to poisoning to fungi on the mealies. (p. 231)

Later, Mitchell (1919) reproduced diplodiosis in cattle by feeding naturally infected maize cobs as well as a *S. maydis* culture grown on sterile maize kernels. Furthermore, diplodiosis has also been recorded in Argentina (Odriozola et al. 2005), Australia (Darvall 1964) and Brazil (Riet-Correa et al. 2013).

In South Africa, diplodiosis is prevalent in late winter where cattle and sheep graze on harvested *S. maydis*-infected maize fields (Kellerman et al. 2005). Under field conditions, diplodiosis becomes apparent from 6 days to 2 weeks after the animals were exposed to *S. maydis*-infected maize cobs and stalks (Kellerman et al. 2005). During experimental reproduction of the disease, where ruminants were fed *S. maydis*-cultured maize kernels, clinical signs were noticeable after a short latent period of 2–8 days (Kellerman et al. 1985). The clinical signs continued for 1–4 days. Initially the animal’s back is slightly arched, there are mild tremors, particularly noticeable over the flank and shoulders, as well as lacrimation and salivation. The animal assumes a wide-based stance and is reluctant to move. Locomotor abnormalities, such as a high-stepping gait, hypermetria and falling, are noticed. These signs are progressive and eventually the animal is unable to rise and becomes paralysed. However, livestock can recover completely if they receive symptomatic and supportive treatment and good nursing care (Kellerman et al. 2005).

Since the first report of diplodiosis by Van der Bijl in 1914, several efforts have been made to isolate the principal toxins responsible for this mycotoxicosis. Different metabolites, namely, diplodiatoxin (Steyn et al. 1972), stenocarpin (Marais 1990), carpellin (Marais 1990), dipmatol (Ackerman et al. 1995), diplonine (Snyman et al. 2011) and chaetoglobosins K, L, M and O (Rogers et al. 2014; Wicklow et al. 2011), have been isolated from *S. maydis*-contaminated maize. A major shortcoming is that none of these *S. maydis* metabolites isolated thus far has been administered to ruminants to reproduce diplodiosis (Masango et al. 2015b). Therefore, the specific role, if any, that the different metabolites play in diplodiosis has not yet been established.

**FIGURE 1:**
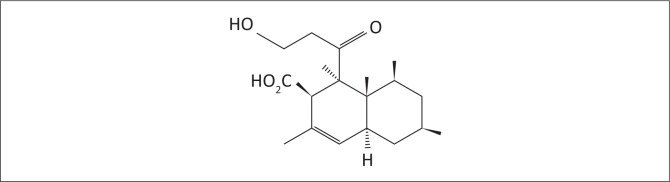
The chemical structure of diplodiatoxin purified from *Stenocarpella maydis*-infected maize culture material.

Diplodiatoxin (C_18_H_28_O_4_) (Figure 1) has initially been isolated and characterised from *S. maydis*-infected maize cultures using bioassay-guided isolation from chloroform–methanol extracts (Steyn et al. 1972).

Furthermore, diplodiatoxin has also been detected in *S. maydis*-infected maize kernels and diseased stalk material as well as from ears collected during a natural field outbreak using nuclear magnetic resonance (NMR) and mass spectrometric data (Rogers et al. 2014; Wicklow et al. 2011). However, diplodiatoxin was reported to account for only 10% of the total toxicity of the maize culture material based on toxicity studies in chickens (Steyn et al. 1972). Diplodiatoxin was reported to induce liver degeneration in chickens (Louw 1969). In acute and subacute toxicity studies in rats, a decrease in body weight and feed intake, dullness, irritability, tremors and convulsions as well as liver damage and inhibition of the brain acetylcholinesterase activity were reported (Rahman et al. 2002; Rao et al. 2003). In *in vitro* toxicity studies, diplodiatoxin induced a concentration-dependent cytotoxicity (Masango et al. 2014) and the accompanying cell death was because of necrosis and caspase-dependent apoptosis (Masango, Ellis & Botha 2015a). The cell death was characterised by mitochondrial damage, cytoplasmic vacuolation and nuclear fragmentation (Masango et al. 2015a).

As diplodiatoxin is considered a major metabolite synthesised by *S. maydis* (Rogers et al. 2014; Wicklow et al. 2011), the aim of this study was to isolate and purify diplodiatoxin from *S. maydis*-infected maize cultures and to administer it to juvenile goats in an attempt to reproduce diplodiosis in a target animal.

## Materials and methods

### Isolation and purification of diplodiatoxin

*Stenocarpella maydis*-infected maize cultures (Coligny 2007 isolate) were prepared by the Grain Crops Institute (ARC-GCI, Potchefstroom, South Africa). Milled culture material (2.3 kg) was soaked in hexane (1.8 L) overnight. The solvent was separated from the culture material using a Buchner funnel and the material was washed with portions of hexane (100 mL/filtration; 1 L in total). The defatted meal was placed in open trays in a fume hood to dry overnight. The dried, defatted maize culture was placed in a plastic bucket with a lid. An ammonia solution was prepared (250 mL of 32% ammonia in 5 L of water) and added to the maize and mixed well to form a slurry. The mixture was left at room temperature for over a weekend. Methanol (2.5 L) was added to the hydrolysate slurry. The mixture was stirred well and left for 1 h with occasional stirring. The liquid was removed by pressing through a mutton cloth (4.2 L obtained). The solution was acidified to pH 2 with concentrated hydrochloric acid (HCl) (200 mL), extracted with chloroform and evaporated to dryness.

The crude extract was purified using flash chromatography. A column was prepared using silica gel (80 mL, Merck silica gel 60, 0.040 mm – 0.063 mm) packed in a glass column (30 mm × 150 mm). The crude extract was dissolved in chloroform (CHCl_3_) and placed on the column and eluted with hexane:ethyl acetate:acetic acid (200:100:12.5). Different fractions were collected and combined according to thin layer chromatography (TLC) analysis. The column was finally eluted with acetone (150 mL). The fraction containing the diplodiatoxin (TLC, Rf 0.42; hexane:acetone:acetic acid [1:4:6 drops]) was re-chromatographed using hexane:acetone:acetic acid (400:100:12.5) to give diplodiatoxin. The compound was recrystallised using ethyl acetate to produce pure diplodiatoxin (pure on TLC). The structure was confirmed with deuterium (^1^H) and carbon-13 nuclear magnetic resonance (^13^C NMR) and compared with literature values (Ichihara et al. 1986; Steyn et al. 1972).

### Animal trial

Three juvenile, weaned Saanen-cross goats weighing between 8 kg and 11 kg were purchased. On arrival the goats were examined to ensure they were clinically healthy. The goats were housed in individual concrete pens at the Onderstepoort Veterinary Animal Research Unit and the study was completed there. The goats had free access to water and were fed a standard ration.

During a 3-week adaptation period the goats were dewormed (Ivermectin; Ivomec Injection, Merial, Halfway House, Gauteng, South Africa), vaccinated against enterotoxaemia (Enterotoxaemia Alum Precipitated Vaccine, Onderstepoort Biological Products, Onderstepoort, Gauteng, South Africa) and received an anticoccidial agent (toltrazuril; Baycox 5%, Bayer Animal Health, Isando, Gauteng, South Africa). Clinical examinations were performed weekly. Blood samples (5 mL serum and 5 mL collecting tube containing ethylenediaminetetraacetic acid (EDTA) as anticoagulant) were collected before and periodically during the trial from the *Vena jugularis*. These were submitted to determine serum enzyme activities (aspartate transaminase [AST] and gamma-glutamyl transferase [GGT]), other chemical pathology parameters (total serum proteins, albumin, urea and creatinine) and complete blood counts. A single goat was dosed at a time and based on the results the subsequent dose level was decided upon before the next animal was used. The dose was estimated based on trials conducted at the Onderstepoort Veterinary Institute (T.S. Kellerman 1984, [ARC-OVI] personal observation). Diplodiatoxin was dissolved in dimethylsulphoxide (DMSO) and administered intravenously (two goats) and intraruminally (one goat). The goats were observed for a minimum of 3 weeks after receiving the last dose.

### Ethical considerations

Ethical clearance was provided by the Animal Ethics Committee, University of Pretoria (project number V123-16).

## Results

The chemical extraction and isolation procedure yielded 370.9 mg of pure diplodiatoxin (Figure 1).

Following intravenous and oral administration of diplodiatoxin to juvenile goats, no clinical signs reminiscent of diplodiosis were observed. The clinical pathology parameters determined fluctuated within normal reference ranges. The dosing regimen and results are presented in Table 1.

**TABLE 1:** Toxicity of diplodiatoxin for juvenile Saanen-cross goats.

No.	Goat		Dosing regimen					Result
	Gender	Age (months)	Body mass (kg)	Dose (mg/kg)	Route	Days dosed	Total dose (mg/kg)	
1	M	4	10	2	IV	5	10	N/a
2	F	4	8	4	IV	5	20	N/a
3	F	5	11	2	Orally	3	6	N/a

M, male; F, female; IV, intravenous administration; N/a, no clinical abnormalities were observed.

## Discussion

The dosage regimen was estimated based on a dosing experiment conducted to determine the toxicity of local *S. maydis* cultures for Boer goats (T.S. Kellerman 1984, personal observation). In Kellerman’s study, three goats each received 5 g of *S. maydis*-infected maize culture material/kg for three consecutive days. All three goats exhibited locomotor abnormalities 3–4 days after dosing commenced. In the current study, the yield of pure diplodiatoxin obtained from 2.3 kg culture material was 371 mg, equating to 0.161 mg diplodiatoxin/g. Thus, 5 g of culture material contained approximately 0.81 mg of diplodiatoxin. The doses administered intravenously to two animals during the current study were 2.5 to 5 times higher and were administered for a longer period. Thus, based on the previous experimental results, the dosage regimen employed in the current study was seemingly appropriate to induce clinical signs. Although individual variation in susceptibility or breed differences might play a role, it seems unlikely that diplodiatoxin is the causative compound.

Snyman et al. (2011) isolated a neurotoxin, diplonine, which induced clinical signs in guinea pigs that were considered as similar to diplodiosis in livestock. However, excessively high oral doses were required to induce neurological signs. Doses equivalent to 200 g culture material/kg and higher were administered. These doses are unphysiological and livestock will not be able to ingest these massive amounts of maize cobs and stover. In addition, Masango et al. (2014) compared the in vitro cytotoxicity of diplonine, diplodiatoxin and dipmatol on Neuro-2a, Chinese hamster ovary (CHO-K1) and Madin-Darby Bovine Kidney epithelial (MDBK) cell lines and concluded that diplodiatoxin was the most cytotoxic and diplonine was not cytotoxic.

In the current study, diplodiatoxin was also administered intraruminally via a stomach tube, as it is possible that diplodiatoxin could be bioactivated by rumen microbes to a more toxic metabolite (Kellerman et al. 2005). Although diplodiatoxin was only dosed to one animal at 2 mg/kg for three consecutive days, no clinical signs were noticed.

It is also conceivable that one or more of the other metabolites synthesised by *S. maydis* may induce clinical signs or a combination of mycotoxins might have a synergistic or additive effect. Wicklow et al. (2011) proposed that a mixture of the different chaetoglobosins might be the causative agent. Their supposition is based on disruption of actin polymerisation which may interfere with the myelination process. In experimental studies of *S. maydis*-induced perinatal mortality, spongiform degeneration of myelin is a common feature and it was concluded that the unknown toxin acts mainly on myelin (Kellerman et al. 1991; Prozesky et al. 1994). The hypothesis is also supported by evidence of demyelination of nerves when vervet monkeys (*Cercopithecus aethiops*) were fed *S. maydis*-infected maize culture as part of their daily diet (Fincham et al. 1991).

## Conclusion

It appears as if diplodiatoxin alone is not the causative compound. Other metabolites and/or mixtures of diplodiatoxin with other mycotoxins, when available in sufficient quantities, should also be evaluated.
